# Supplementary material for: A macroscopic violation of no-signaling in time inequalities? How to test temporal entanglement with behavioral observables
Source: Front Psychol. 2015 Jul 29;6:1061. doi: 10.3389/fpsyg.2015.01061 (PMC4518645; doi:10.3389/fpsyg.2015.01061)
Supplement: Supplementary file 1 [file Data_Sheet_1.DOCX]

Supplementary Material

Raw coincidences of the three experiments and their joint probabilities following the *NSIT* formalism presented in equation 1.

Table 1: Raw coincidences observed in Experiment 1:

|  | **Key pressed** | | |  |
| --- | --- | --- | --- | --- |
| **Picture** | **LEFT+** | **RIGHT-** | Total |  |
| **LEFT+** | ***1939*** | 1391 | 3330 |  |
| **RIGHT-** | 1844 | ***1486*** | 3330 |  |
|  |  |  |  |  |
| **P(Q_2= +1)=** | P(Q_1= -1, P(Q2=+1) | + | P(Q_1= +1, P(Q2=+1) | ∆ |
| 0.500 | 0.277 | + | 0.291 | **-0.0680** |
| **P(Q_2= -1)=** | P(Q_1= +1, P(Q2=-1) | + | P(Q_1= -1, P(Q2=-1) |  |
| 0.500 | 0.209 | + | 0.223 | **0.0680** |

Table 2: Raw coincidences observed in Experiment 2:

|  | **Key pressed** | |  |  |
| --- | --- | --- | --- | --- |
| **Picture** | **LEFT+** | **RIGHT-** | Total |  |
| **LEFT+** | ***3099*** | 981 | 4080 |  |
| **RIGHT-** | 3114 | ***966*** | 4080 |  |
|  | | | | |
| **P(Q_2= +1)=** | P(Q_1= -1, P(Q2=+1) | + | P(Q_1= +1, P(Q2=+1) | ∆ |
| 0.500 | 0.382 | + | 0.380 | **-0.2614** |
| **P(Q_2= -1)=** | P(Q_1=+1, P(Q2=-1) | + | P(Q_1= -1, P(Q2=-1) |  |
| 0.500 | 0.120 | + | 0.118 | **0.2614** |

Table 3: Raw coincidences observed in Experiment 4:

|  | **Key pressed** | | |  |
| --- | --- | --- | --- | --- |
| **Picture** | **LEFT+** | **RIGHT-** | Total |  |
| **LEFT+** | ***4910*** | 5024 | 9934 |  |
| **RIGHT-** | 4904 | ***4773*** | 9677 |  |
|  |  |  |  |  |
| **P(Q_2= +1)=** | P(Q_1= -1, P(Q2=+1) | ***+*** | P(Q_1= +1, P(Q2=+1) | ∆ |
| 0.500 | 0.25 | ***+*** | 0.25 | **0.000** |
| **P(Q_2= -1)=** | P(Q_1= +1, P(Q2=-1) | + | P(Q_1= -1, P(Q2=-1) |  |
| 0.500 | 0.256 | + | 0.243 | **0.000** |

Table 4: Raw coincidences observed in all three experiments

|  |  | **Key pressed** | |  |
| --- | --- | --- | --- | --- |
| **Picture** | **LEFT+** | **RIGHT-** | Total |  |
| **LEFT+** | ***9948*** | 7396 | 17344 |  |
| **RIGHT-** | 9862 | ***7225*** | 17087 |  |
|  |  |  |  |  |
| **P(Q_2= +1)=** | P(Q_1= -1, P(Q2=+1) | + | P(Q_1= +1, P(Q2=+1) | ∆ |
| 0.500 | 0.286 | + | 0.289 | **-0.0754** |
| **P(Q_2= -1)=** | P(Q_1= +1, P(Q2=-1) | + | P(Q_1= -1, P(Q2=-1) |  |
| 0.500 | 0.215 | + | 0.210 | **0.0754** |
